# Women and men in orthopaedics

**DOI:** 10.1051/sicotj/2021020

**Published:** 2021-03-26

**Authors:** Costantino Errani, Shinji Tsukamoto, Akira Kido, Azusa Yoneda, Alice Bondi, Frida Zora, Fotini Soucacos, Andreas F. Mavrogenis

**Affiliations:** 1 Department of Orthopaedic Oncology, IRCCS Istituto Ortopedico Rizzoli 40136 Bologna Italy; 2 Department of Orthopaedic Surgery, Nara Medical University 634-8521 Nara Japan; 3 Department of Rehabilitation Medicine, Nara Medical University 634-8521 Nara Japan; 4 European University of Cyprus 2404 Nicosia Cyprus; 5 First Department of Orthopaedics, National and Kapodistrian University of Athens, School of Medicine 157 72 Athens Greece

**Keywords:** Orthopaedics, Women, Specialty, Gender

## Abstract

*Purpose*: To compare and discuss the gender disparities in the Orthopaedic specialty. *Methods*: We reviewed the literature to find the rates of women applying for an orthopaedic residency, fellowship, and academic career program, to understand the causes of the disparities in women in orthopaedics, and how this relates to orthopaedic surgical practice. *Results*: The idea that men and women are different and have different working styles and skills and the belief that males are more dominant and more status-worthy than females leads to gender barriers and stereotypes that restrict women from entering male-dominated specialties. It is important to mention that equivalent barriers restrict men from pursuing female-dominated specialties such as Gynecology. Economic disparities and gender stereotypes that divide medical specialties into masculine and feminine, creating a gender gap in health care are major concerns. However, the number of women in the health sector is expected to increase due to the growing amount of female students that are expected to soon graduate. A leadership gender gap also exists; although women consist of 70% of the health care workforce they occupy only 25% of leadership positions. *Conclusion*: The existence of gender-based disparities in healthcare is multifactorial. The explanation behind the existence of a so-called gender gap lies in organizational and individual factors. Early development and family relations, the decision between work and life balance, personal choices and interests, as well as working conditions, absence of role models and mentorship and institutional policies make gender disparities even more evident.

## Women in medicine and surgery

Elizabeth Blackwell (1821–1910) was a British physician, notable as the first woman to receive a medical degree in the United States, and the first woman on the Medical Register of the General Medical Council [1]. She pioneered promoting education for women in medicine. Initially a schoolteacher, she found interest in medicine after a friend fell ill and remarked that, had a female doctor cared for her, she might not have suffered so much [1]. She began applying to medical schools but she was rejected from every medical school she applied to because of gender disparities. In October 1847 she was accepted as a medical student at the Geneva Medical College in New York. On January 23, 1849, she became the first woman to achieve a medical degree in the United States [2, 3]. In Europe, the first women in medicine were Elizabeth Garrett Anderson (1836–1917), Mary Corinna Putnam Jacobi (1842–1906), and Madeleine Alexandrine Brès (1842–1921). Elizabeth Garrett Anderson was the first woman to qualify in Britain as a physician and surgeon; she studied medicine at the University of Sorbonne in Paris where she obtained her medical degree in 1870 [4]. Mary Corinna Putnam Jacobi was also British; she studied Pharmacy in Philadelphia and medicine at the Paris School of Medicine. In 1871, she became the second doctor of the Faculty of Medicine in Paris, after Elizabeth Garrett Anderson [5]. Madeleine Alexandrine Brès studied medicine at the University of Paris and she was the first French woman to obtain a medical degree in 1875 [6].

The first women in surgery were Marie Nageotte-Wilbouchewitch (1864–1919) and Emily Dunning Barringer (1876–1961). Marie Nageotte-Wilbouchewitch was born in Russia and studied medicine at the Paris Medical School. She was the first woman to complete the medical internship program, and in 1893 she graduated as a doctor of medicine. She was a pioneer in pediatric orthopaedics, and wrote numerous articles and book chapters including the “Atlas-manual orthopedic gymnastics, treatment of waist deviations (1903)”, “Kinesitherapy, massage, mobilization, gymnastics (1909)”, and “Treatment of spinal deviations and respiratory failure (1937)” [7]. Emily Dunning Barringer was the first female ambulance surgeon and the first woman to be accept accepted for an internship and to complete a surgical residency. She completed her residency at the Gouverneur Hospital in New York City in 1904 [8].

Women and men practice medicine differently. Surgical disciplines are disproportionately male despite increasing numbers of female medical students [9–15]. Similarly, Orthopaedics is and will probably remain a male-dominated specialty [16]. The percentage of female residents in orthopaedics has not changed despite the increase in female representation that is seen in other specialties of medicine [16]. Orthopaedic specialty has the lowest percentage of women residents (15.4%) and faculty of any other medical specialty [17–19]. In 2010 in USA, women represented 47.8% of medical students, but only 13.2% of orthopaedic residents, 6.5% of the AAOS members (0.6% in knee society and 0.6% in hip society), and 8.7% of academic positions within USA orthopaedic surgeons [20]. In France, among 3553 orthopaedic surgeons, there were approximately 248 (7%) women in 2019; in Greece, among 2226 orthopaedic surgeons there were approximately 68 (3%) women in 2020; in Italy, among 4434 orthopaedic surgeons there are approximately 456 (10.2%) women at the time of this writing; and in Japan, women orthopaedic surgeons account for approximately 4% of all the Japanese Orthopaedic Association (JOA) members. Representation of women in orthopaedic research is not much different; women faculty represent only 12% of all orthopaedic, a disparity that has been found to be independent of numbers of publications [17, 21]. Fewer women apply to fellowships and a smaller percentage of women pursue careers in orthopaedics than in other specialties with the lowest representation of women in leading positions [18, 22]. Larson et al. found a significant underrepresentation of women, overall in 2013 and 2015, as keynote speakers, plenary speakers, and invited lectures at medical society conferences [23]. Ence et al. [24] performed a survey for faculty at 142 civilian academic orthopaedic departments in the United States. They found that although women had a lower Hirsch index (*h*-index, defined as the number [*h*] of an investigator’s publications that have been cited at least *h* times) and shorter career durations than their male counterparts overall, they had a similar *m*-index, which is defined as the *h*-index divided by academic career duration in years, suggesting that the observed *h*-index disparities were due to differences in career duration. They also found that gender had no independent predictive value for ranking among academic orthopaedic surgeons; women were found to have shorter careers than male surgeons, with career duration correlating with academic rank. This could explain the relative paucity of women in leadership positions in academic orthopaedic departments [24].

At the authors’ institutions and possibly worldwide, more women are currently applying for an orthopaedic residency. But is there any evidence for the need to increase women in orthopaedics [16]? The hunt for differences between men’s and women’s brains is full of poor research practice and biases mediated by feminism and masculism, and media [25]. Neuroscientific research on sex difference is currently a controversial field, frequently accused of purveying a neurosexism that functions to naturalize gender inequalities. Additionally, neuroscientific research on sexual dimorphism has recently elicited intense criticism from scholars in both natural and social sciences. These critics contend that the evidence-based for many claims of sex difference is plagued by bias and methodological weakness [26–31]. The debate on gender-appropriate research must not only take into account neuroscientific knowledge production but also the transformation of neuroscientific facts into the public via social media [32, 33].

## Gender diversity in surgery

The benefits of gender diversity have been reported previously [17, 34, 35]. In business, a correlation has been drawn between improved financial performance and gender parity in leadership roles [36]. In medicine, gender diversity has been shown to improve patients’ outcomes and satisfaction [17]. Previous studies showed that female physicians seem to have more empathy and sensitivity than their male counterparts, spending more time with patients and getting to mutual decision-making [14, 37]. However, how does this relate to orthopaedic surgical practice? Dineen et al. showed that patients have not preferenced for the gender of their orthopaedic surgeons. However, there is evidence that suggests that patients are more comfortable with men surgeons and/or physicians of their same gender [16]. Canada found that women have a higher proportion of match success to orthopaedic fellowship when compared with men applicants [22].

Surgery has a major technical component, so there is less reason to expect a difference in outcomes between female and male surgeons [38]. Wallis et al. conducted a retrospective study of patients having surgical procedures from all surgical specialties in Ontario, Canada, between 1st January 2007 and 31st December 2015, to assess the hypothesis that the gender of the operating surgeon would significantly affect 30-day postoperative outcomes. They identified 1,159,687 eligible patients. Surgery was performed by 3314 surgeons, 774 (23.4%) of whom were female, and 2540 (76.6%) were male. Female surgeons performed proportionally more operations than men in general surgery, obstetrics and gynecology, and plastic surgery. They found that patients treated by female surgeons had a statistically significant decrease in 30-day mortality, yet with no difference in readmissions or complications, and similar surgical outcomes compared with those treated by male surgeons. However, more patients treated by female surgeons had their operation in later calendar years. Additionally, differences in a surgical specialty, procedural volume, and age of female surgeons meant that patients in primary analysis who were successfully matched to male surgeons were treated by younger surgeons with less experience and lower annual volumes than those who were excluded. Moreover, included patients were more likely to have had general surgery or an obstetrics and gynaecology related procedure and less likely to have had neurosurgery, orthopaedic surgery, or a urology-related procedure. Improved postoperative outcomes for patients treated by female surgeons were restricted to patients who had elective operations, which might reflect better patient selection for surgery or residual confounding; patients who had emergent procedures were less likely to be female and more likely to have these procedures performed by younger surgeons with lower surgical volumes who had practiced for a shorter period of time [38].

## Orthopaedic disparities

Understanding the causes of the disparities in women in orthopaedics is the first step to draw useful solutions [36]. Rohde et al. analyzed the possible reasons for why women might not choose orthopaedics and they made a survey to 232 resident members of the Ruth Jackson Orthopaedic Society. Questions were formulated to determine the demographics, practice patterns, and lifestyle choices of women who chose orthopaedic surgery as a specialty. The most common specialties among respondents were hand (24%), general orthopaedics (20%), pediatric orthopaedics (19%), and sports (15%). The most common reasons cited for having chosen orthopaedic surgery were enjoyment of manual tasks (71%), professional satisfaction (54%), and intellectual stimulation (53%). The most common reasons proposed for why women might not choose orthopaedics included factors such as physical strength and perceived lack of work-life balance as deterrents for females entering orthopaedics [18]. Hariri et al. e-mailed a self-administered survey to 498 U.S. orthopaedic residents, querying them about their fellowship specialty choice and their career plans: 430 respondents (86%) were male, sixty-three (13%) were female, and five (1%) did not provide information regarding sex. They reported that significantly more women than men were planning on pursuing a pediatric fellowship (24% vs. 6%, respectively) and significantly fewer were planning on pursuing a sports fellowship (11% vs. 31%, respectively). Significantly more women than men plan on a subspecialty-only practice (62% vs. 34%, respectively). The projected retirement age of sixty-four years for current residents is roughly equal to that of the previous generation. There was no difference between men and women with regard to leadership and research aspirations, projected retirement age, and projected workdays per week. However, significantly more women than men (65% vs. 47%, respectively) planned on reducing their work hours or changing to part-time status at some time during their careers. There is a higher percentage of female residents (13%) than female practicing orthopaedists (4%) in the United States. Given the trend toward an increasing proportion of female orthopaedists and the higher likelihood that they will reduce their work hours during portions of their career, policymakers should consider training more orthopaedists to ensure patient access to timely, quality orthopaedic care [39]. Amoli et al. [40] performed the 2015 Pediatric Orthopaedic Society of North American (POSNA) Needs Assessment Survey for POSNA members and a special 36-question survey for recent pediatric orthopaedic fellowship graduates. Among the new graduates, women were more likely to choose an academic practice, whereas men were more likely to choose private practice. The primary reasons for choosing a job were not different between men and women. Among the new graduates, geography/family considerations were reported as being highly important when selecting a job followed by academic opportunities. Interestingly, a higher percentage of males reported finances as being important when selecting a job. For the current POSNA members, the most important reasons when choosing a job for both men and women were quality of partners and interesting practice. There was no difference in starting salaries between men and women. When stratified by practice type, for private practice starting salaries, over half of men placed in the highest category of >USD 400,000, whereas the single woman respondent placed in the lowest category of <USD 300,000. Men were more likely to report having job offers before starting their fellowship. Finally, among POSNA members, women reported a lower weekly surgical case volume compared with men. Of the men, 108 of 408 (26%) reported performing more than seven surgeries per week compared with 12 of 122 women (10%). More men than women planned to reduce their workload or retire in the next 5 years [40]. As a result of a survey for the 90 residents at one residency program, Pico et al. [41] reported no differences between males and females’ performance. Although females pursue orthopaedic residency less frequently than males, performance during residency should not bias their future selection. Whitaker et al. [42] reported, by surveying a cohort of medical students at a single institution, that both male and female students ranked “work-life balance” and “variety in specialty” among the top three most important preferences. Females ranked “range of practice options,” higher than males, and males ranked “previous exposure to the specialty” higher than females. Both males and females indicated that orthopedics is “male-dominated,” has “competitive entrance requirements,” and requires “long residency work hours.” They differed in their perception of “requires physical strength” (60% females vs. 38% males), and by how much orthopaedics is “male-dominated” (95% females vs. 77% males). The first latent explanatory factor for females consisted of “work-life balance,” “residency length,” “residency work hours,” and “family-friendly specialty.” Although the first latent factor for males consisted of “prestige,” “income potential,” “grade or step scores,” and “competitiveness of residency program.” By identifying the multifactorial areas that may be inadvertently discouraging females from applying, orthopaedic residency programs may be able to better address those issues and attract the best talent of both genders [42]. For example, it is important to teach female medical students that orthopedic surgery has greatly progressed from the brute force discipline of the past, with new techniques and equipment decreasing the strength requirement [43]. B. Shubin Stein, MD, assures women that “with the right technique and the right tools, I can do everything the boys can do” [43]. Hill et al. tried to understand how orthopaedic surgery has been less successful in recruiting women compared with general surgery: a questionnaire survey was e-mailed to 529 orthopaedic residents by the American Academy of Orthopaedic Surgeons. They reported that acceptance by a senior faculty was a barrier for a female who wants to become orthopaedic surgeons [20]. Bucknall and Pynsent reported that male domination was one of the reasons influencing negatively the career choice in orthopaedic surgery for female medical students [44]. Several studies reported that the lack of mentorship and women faculty in training programs is a possible cause of smaller numbers of female medical students who choose a career in orthopaedic [18, 45–48]. The programs with the most female residents were found to have greater numbers and percentages of female faculty and women in leadership positions, suggesting greater availability of same-sex mentors for female applicants [49].

In order to attract the most capable medical students to our specialty, we are willing to increase the number of women who practice orthopaedic surgery [36]. It is important that we prove to medical students that life as an orthopaedic surgeon and raising a family are not mutually exclusive pursuits [36]. Baldwin et al. [50] found that 78.9% of men and 79.1% of women thought that a career in orthopaedic surgery would be more difficult for a woman to balance with a family. Nevertheless, many successful female orthopaedic surgeons have families. Promoting these women may address this concern by not only increasing the number of role models available to female medical students and residents but also acknowledging the value that female orthopaedic surgeons with families add to the profession. Mason et al. found that orthopaedic internship programs had a positive impact on increasing the odds of each student participant applying to an orthopaedic surgery residency program [51]. Hill et al. reported that increased exposure to orthopaedic content during medical school and increased female mentorship may help recruit more women into the orthopaedic surgery workforce. By comparing orthopedics with general surgery, resident women reported that more peers choose to go into general surgery instead of orthopedics due to greater acceptance by the senior faculty in that field [20]. Early exposure of female medical students to orthopaedics during medical school can help correct this gender disparity, as well as promoting female role models and females in leadership roles [16–18, 52–54]. Programs designed to improve scholarship and mentorship of medical students could increase interest in our specialty, overcoming some barriers and providing a path for more diversity [17, 18]. By increasing the number of women residents, the percentage of women fellowship could increase [22]. To increase the number of women residents, a program’s website is more important than printed materials from a program. This is not unexpected as the new generation of surgeons turns to electronic media for information as it is readily accessible. Programs may find this interesting so that they may create comprehensive web pages [55]. When deciding which orthopaedic surgery residency programs to rank the highest, Goss et al. found that the most important factors for female applicants included camaraderie among current residents, the happiness of current residents, variety and number of cases, successful placement of recent graduates into desired subspecialty fellowship, and early surgical/clinical experience. To increase sex diversity in their residency programs, the focus should be placed on the current residents themselves highlighting their happiness within the program and friendship with their co-residents during interview days and on program websites. Ensuring up-to-date case logs, alumni profiles including fellowships, and accurate schedules showcasing the amount of time spent in the operating room early in residency on program websites may also help orthopaedic residency programs to increase their sex diversity [56].

Achieving gender equity in international society meetings and academic faculty roles can help reduce the gender difference within our specialty [22, 23, 54, 57, 58]. A collaboration between medical schools and medical societies might play an important role in supporting the careers of women [58]. The aim is to attract to orthopaedics not only the most capable male medical students but also the most capable female medical students with equal rights as well as responsibilities, surgical practices, skills, and academic profile.

## Socioeconomic and cultural disparities

Economic disparities and gender stereotypes that divide medical specialties into masculine and feminine, creating a gender gap in health care are concerns that are imperative to be discussed and solved now more than ever. “Men and women will achieve paying equality in 257 years” according to data that was published by the World Economic Forum in 2019 [59]. That is 55 years more than in their previous estimation in 2018. Specifically, in the health care sector, women are often underpaid or even unpaid. Evidence shows that they contribute 5% to the global gross domestic product (GDP), out of which 50% is unrecognized and unpaid [60]. It is quite peculiar that the paying gap in men’s favor is nearly universal and unexplained. Possible factors include age, experience, education, number of hours worked, or specialty choice all of which suggest discrimination and bias against women and in favor of men [59, 60]. According to the Organisation for Economic Co-Operation and Development (OECD), the percentage of female doctors has been increasing exponentially for the last fifteen years and women represent close to 70% of the Global Healthcare Workforce [61, 62]. It is important to mention that although females constitute such a big percentage, they are mostly concentrated in midwifery and nursing professions while far fewer are physicians [60].

The number of women in the health sector is expected to increase even more due to the growing amount of female students that are expected to soon graduate and join the global health care workforce [63]. Even though these statistics support the existence of gender-based economic disparities the issue is overlooked. As mentioned above this could be the case due to the feminization and masculinization of certain specialties accordingly which often results in bias. According to the AAMC, out of the 43 specialties featured in the Physician Specialty Data Report for the year 2015, only Gynecology and Pediatrics are female dominant specialties. It is very interesting to notice that female doctors in Gynecology consist 52% when at the same in Orthopaedics they consist only 5% [64]. According to the WHO, occupational segregation is affected mostly by socioeconomic and cultural factors [60]. It has been proven through studies that the leading cause of occupational segregation is the gender stereotype that considers men to be the breadwinners and depicts women as the homemaker and child carer [65, 66]. This limits women when it comes to decision-making regarding which specialty to follow or pursuing leadership positions. During the years 1991–1995, it was found that male students were almost three times more likely than their female colleagues to pursue a surgical residency. The same study also showed that women were 2.1 times more likely to follow Gynecology than men [67]. This translates into gender essentialism and male primacy [60].

The idea that men and women are different and have different working styles and skills and the belief that males are more dominant and more status worthy than females leads to gender barriers and stereotypes that restrict women from entering male-dominated specialties while it is very important to mention that equivalent barriers restrict men from pursuing female-dominated specialties such as Gynecology [68]. Men and women also spend quite a different amount of time on unpaid care work, with women spending between 2 and 10 times more time on unpaid care compared to men, depending on the country [60, 69]. In general, women carry out almost three more hours of unpaid work per day than men [60, 70, 71]. The Commission on women and health in 2015 analyzed data that showed the female financial contribution amounted to nearly 5% of global GDP [60]. Unpaid and informal work on behalf of women makes up nearly half of their economic contributions to the health care system [60, 72]. The issue rises due to the fact that although women contribute to the health care system’s well-being, it often goes unrecognized and unaccounted for in decision making [60]. In Spain, 88% of all health care work is unpaid [73].

A leadership gender gap is also observed. Although women consist of 70% of the health care workforce they occupy only 25% of leadership positions. According to the WHO statistics, only 31% of ministries of health globally are led by women. It is evident that decision-making remains in the hands of men with 69% of organizations and 80% of organization boards being led by men [74]. At the high end is the Africa Region with 38%, and South- East Asia at the low end with 18% of ministries of health led by women [60]. The difference in representation varies between different specialties [68]. One study concluded that women received only 1 in 10 awards in health and medicine, while another study found that female managers felt that their voices were not as respected as those of their male colleagues and also faced discrimination due to their younger age [75, 76]. Another study proved that both women and men have a subtle bias towards women when it comes to hiring and promoting them [77]. These biases increase gender gaps in the health care sector. For example, women represent only 20% of deans in the top 25 global schools of medicine and 36% in the top 25 global schools of public health [77].

Of particular interest is the case of Greece. According to the European Institute of Gender Equality progress report, Greece descendent to the lowest rank in the Gender Equality Index (50.0), is the only EU country with a deteriorating score over a 10 year period in the domain of economic and social power of women. Greece was also ranked low in women’s representation in medicine amongst OECD countries out of 65,499 doctors 27,549 were women, meaning 41.20% when at the same time is estimated that only 11% of them follow an academic career [62, 78–81].

The existence of the above gender-based disparities in healthcare is multifactorial. Through research and evidence-based processes some factors have been identified. According to the WHO, the explanation behind the existence of a so-called gender gap lies on organizational and individual factors. Early development and family relations, the decision between work and life balance, personal choices and interests, as well as working conditions, absence of role models and mentorship and institutional policies make gender disparities even more evident [60].

## Conflicts of interest

All authors declare no conflicts of interest.
